# Nano-Scaled Materials and Polymer Integration in Biosensing Tools

**DOI:** 10.3390/bios12050301

**Published:** 2022-05-05

**Authors:** Hichem Moulahoum, Faezeh Ghorbanizamani, Emine Guler Celik, Suna Timur

**Affiliations:** 1 Biochemistry Department, Faculty of Science, Ege University, Bornova, 35100 Izmir, Turkey; hic_moul@hotmail.com (H.M.); faezeh.zamani2@gmail.com (F.G.); 2 Bioengineering Department, Faculty of Science, Ege University, Bornova, 35100 Izmir, Turkey; emine.guler.celik@ege.edu.tr; 3 Central Research Testing and Analysis Laboratory Research and Application Center, Ege University, Bornova, 35100 Izmir, Turkey

**Keywords:** nanocomposites, polymer scaffolds, nanoparticles, optical sensing, point-of-care diagnostics

## Abstract

The evolution of biosensors and diagnostic devices has been thriving in its ability to provide reliable tools with simplified operation steps. These evolutions have paved the way for further advances in sensing materials, strategies, and device structures. Polymeric composite materials can be formed into nanostructures and networks of different types, including hydrogels, vesicles, dendrimers, molecularly imprinted polymers (MIP), etc. Due to their biocompatibility, flexibility, and low prices, they are promising tools for future lab-on-chip devices as both manufacturing materials and immobilization surfaces. Polymers can also allow the construction of scaffold materials and 3D structures that further elevate the sensing capabilities of traditional 2D biosensors. This review discusses the latest developments in nano-scaled materials and synthesis techniques for polymer structures and their integration into sensing applications by highlighting their various structural advantages in producing highly sensitive tools that rival bench-top instruments. The developments in material design open a new door for decentralized medicine and public protection that allows effective onsite and point-of-care diagnostics.

## 1. Introduction

Developing new materials and exploiting analytical devices to determine, monitor, control, and quantify specific molecules in the environment and the human body has become necessary in biosensing. Clark and Lyons first introduced biosensors in 1962 when an oxygen electrode was used to selectively detect glucose levels [1]. Since then, numerous studies have focused on improving these tools by developing efficient biosensing devices to detect a variety of other analytes and, more specifically, trace-quantity analytes. Nowadays, biosensors are widely used in biomedicine and health, environmental monitoring, drug development, forensics, and food safety. Despite all the successes in this area, developing more sensitive and selective devices that detect low target concentrations via effective transducing elements and recognition materials is challenging. The selectivity and sensitivity of the biosensing devices can be directly or indirectly affected by the preparation of analyte samples. Some analytes are now detected without the need for preparation procedures. For example, hazardous chemicals and heavy metals such as mercury can be directly traced without sample preparation in contaminated water [1].

Conversely, other samples necessitate intricate multistep preparation procedures to minimize interferences and enrich the target molecules to reach detectable concentrations and enhance analytical performances. Moreover, the presence of interferents and biofouling are two main problematic issues that directly influence the performance of biosensors. Electroactive substances interfere with the results when analytical measurements are made on physiological materials such as blood. For example, interferents such as ascorbate or acetaminophen (paracetamol) can negatively affect the glucose sensors as these substances are oxidized at the electrode surface. Biological fluids such as blood can deposit biomolecules (i.e., proteins) which eventually block the surface area. In this case, the surface biofouling blocks the electron passage through the analyte to the surface and reduces the response signal [2].

Robust automated systems containing advanced materials can significantly enhance analytical performance. The recent development of polymer science has made significant progress in sensitivity and selectivity enhancement, response time shortening, and flexibility increase for the immobilization of biomolecules to biosensor platforms. The earliest polymer structures utilized in biosensing devices were the fluorinated ionomer Nafion [3] and cellulose acetate [4]. After their initial success, a range of other polymers was employed to modify the recognizing surfaces and prevent their biofouling [5]. Polyethylene glycol (PEG) and polyethylene oxide (PEO) are the most commonly used polymer materials [5]. These biocompatible composite polymers are highly soluble in aqueous systems allowing them to mimic the typical conditions found within biological systems. Polymeric structures such as dye-loaded polymersomes can be used successfully to detect illegal drugs (cocaine, methamphetamine, synthetic cannabinoids, etc.) in various bodily fluids, including saliva and urine [6,7].

Along with the advances in polymer science, there has been a clear transition from using their insulating features toward their conductive properties [8]. Many sensor platforms were designed using polymer materials, including planar polymers, vesicular polymers, polymersomes, hydrogel materials, conducting polymers, and molecularly imprinted polymers. However, despite the improvements in polymer materials’ employment in biosensing platforms and their commercialization, the need to improve current systems and their analytical performances is still a subject of interest.

Nanomaterials and nanotechnology have received tremendous interest and have become leaders in analytical chemistry over the last decades. Desirable nanoparticles properties, such as the ability to tailor size, structure, and surface-to-volume ratios, provide excellent possibilities for designing novel sensing systems and enhancing the performance of the current bioanalytical assays. Combining nanomaterials with polymers through physical/chemical crosslinking to polymeric chains leads to nanocomposite polymers with new exclusive properties. The present review encloses a specific overview of these polymeric nanocomposite materials and their impact and integration in biosensing and diagnosis applications. Nanocomposite polymers are a relatively rising material niche with several promising applications. This review takes a broader look at the general sensing abilities of these materials with a significant focus on non-invasive approaches in biomedical applications from our own experiences and the many reports found in the literature. While the combination of polymers and nanomaterials provides many options, the current manuscript mainly focuses on planar and vesicular polymer nanocomposites, hydrogel materials, conducting polymers, and molecularly imprinted polymers in biosensors design.

## 2. Polymer and Biopolymer Nanocomposites

Polymers have been considered prominent candidates for creating an ideal matrix for entrapment and immobilization of biomolecules in the analytical sciences. Characteristics of most polymers, such as high conductivity, ease of biofunctionalization, flexibility, biocompatibility, highly modifiable chemical functions, etc., make them attractive for biosensor development in different fields from environmental analysis to biomedical applications [9]. Our experience with polymeric and co-polymeric materials also demonstrates the successful application of functional polymers for the immobilization of enzymes [10], micro-organisms [11], antibodies [12], and aptamers [13] in the design of electrochemical biosensors. Historically, polymers have been seen as a practical material choice for electrochemical devices, but recent advances in nanotechnology and the creation of nano-scaled materials has allowed for even further evolution in the biosensor field development due to new intrinsic optical, electrical, and mechanical properties.

Efficient immobilization of bioreceptors (or other components) and optimal signal transduction are crucial for biosensors. Polymers are still key coating matrices for nanomaterials through the fusion of nano-objects and polymers. These combinations have led to the emergence of new nano-scaled hybrid materials or polymer nanocomposites. They have been further employed to construct polymer nanocomposite-based biosensors to obtain highly sensitive and reliable analytical devices by improved catalytical and chemical reactivity, surface specificity, enhanced electrode kinetics, controllable synthesis and morphologies, higher stability, and biocompatibility [14]. As an alternative to synthetic polymers, some features of biopolymers, such as natural origin, biodegradability, recyclability, lower antigenicity, and their suitable interaction with living systems, make them powerful tools for biosensor fabrication [15,16].

Polymeric and biopolymeric nanocomposites refer to a hybrid structure in which a polymer matrix is used as a substrate, and nano-scaled organic or inorganic materials are used as fillers. Typically, the polymers of poly(lactic acid) (PLA), poly(ethylene oxide) (PEO), poly(lactic-co-glycolide) (PLG), poly(N-isopropyl acrylamide) (PNIPAM), and polyurethanes, etc. have been utilized as the matrix phase of polymeric nanocomposites. Biopolymeric nanocomposites, which are also called “Bio-nanocomposites”, “bio-hybrids”, and “green nanocomposites” by the popular terms of recent years, are made up of a nanosized additive in naturally occurring polymers including cellulose, chitin, collagen, silk, keratin, alginate, lignin, starch, polyhydroxyalkanoates (PHA), etc. [17]. The merging of nanosized filler materials into the polymeric matrix produces interesting and improved mechanical, thermal and optical properties. According to this reinforcement strategy, it can be said that filler materials act as molecular bridges enhancing and controlling dimensional stability, flexibility, strength, toughness, durability, thermal stability and conductivity, optical properties (color and transparency), size, distribution, and shape [15,18,19]. Organic materials (carbon nanotubes (CNTs) and graphene) and inorganic materials (silicates and metal/metal oxides) are the kinds of nanofillers used to prepare nanocomposites made of polymers [18,19]. The characteristics of polymeric nanocomposites are affected by choice of both the filler and matrix. For instance, while the type of polymer matrix significantly determines the hydrophobicity, transparency, strength, toughness, controlled ionizability, crystallinity, functionality, biocompatibility, and biodegradability, the choice of filler considerably affects the structural and functional properties. Hence, unique polymer nanocomposites can be synthesized by various combinations of nanofillers. This diversity provides application-oriented strategies via selection of filler nano-objects for the desired properties of specific fields, including medicine, diagnostics, biomedical applications, food packaging, optoelectronic devices, biosensing, bioimaging, tissue engineering, cosmetics, energy, etc. [17,20].

In parallel to their flexible functionalities and fascinating properties, polymeric nanocomposites have been extensively studied to improve sensor performance and have remarkably allowed the fabrication of many novel biosensors in recent years [21,22,23]. For example, while quantum dots–polymeric nanocomposites exhibit excellent fluorescence properties that can be used in optical biosensors, CNTs–polymeric nanocomposites provide significant enhancement in the mechanical property that can be adapted to an optoelectronic sensing device [20]. The utilization of polymeric nanocomposites provides needs-based designs. It brings additional key performance parameters, including higher sensitivity and selectivity, lower detection limits, good reproducibility, and stability by providing a large and easily adjustable surface area, higher electrical conductivity, and fast electron transfer rate [23].

For polymeric nanocomposites, chemists and material scientists have described various synthesis methods, including ion exchange, template synthesis, sol-gel, in-situ polymerization, hydrothermal route, melt intercalation techniques, etc. [20,24]. The successful design of a polymeric nanocomposite with any required property is a critical step toward the control of interfacial interactions between the nanofiller and the polymer matrix. Understanding the influence of the filler on the size, shape, orientation, dispersion, and compatibility of the polymer matrix is the most important consideration. When creating a new polymer nanocomposite material, an effective formulation is required by considering three main approaches: rationality-based design, functionality-based design, and tailored property-based design. In the design of polymeric nanocomposites, process route, temperature, pressure, and time are the parameters required to be controlled during processing. The nanofiller choice needs to consider the filler shape, size, type, volume, weight, and orientation. In contrast, the matrix preparation needs to consider the kind of polymer, surface nature, and chemistry. Based on the above, the combination of the nanofiller and the polymer matrix must be achieved at a nanoscale level with chemical compatibility and homogenous dispersion.

**Figure 1:**
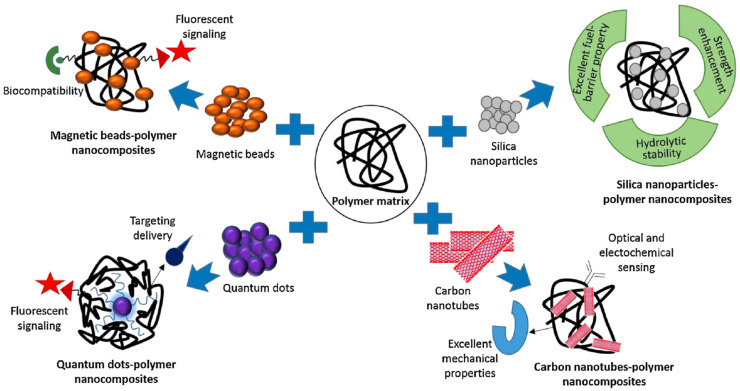
Effect of nanofiller materials on polymer nanocomposite properties. Reprinted with permission from ref. [20]. ©2018, Elsevier.

Polymer nanocomposites are microstructures of hybrid organic–inorganic materials that can be formed into three types; unintercalated (or microcomposite), intercalated (and/or flocculated), or exfoliated (or delaminated). These microstructural forms are controlled by the synthesis method. Of these various synthesis methods, the melt-blending method is eco-friendly because of the lack of solvent usage and is industrially scalable due to its cost effectiveness, however the need for a high temperature that can damage the surface of nanofiller is its main disadvantage. On the other hand, the in-situ polymerization technique provides better exfoliation in comparison to the melt intercalation method. In the case of sol-gel technology, disadvantages such as high temperature, which can cause the degradation and aggregation of polymers, make it uncommon. There are different synthetic methodologies including organic treatment and chemical modifications for the polymer nanocomposites manufacturing process [25]. Click chemistry and ring opening of epoxides and aziridines are very efficient and common chemical concepts in the fabrication of polymer nanocomposites. In particular, click reactions are versatile coupling methods due to advantages that include methodological simplicity, high reaction yield and high reaction rates, moderate reaction conditions, easily removable byproducts etc. CuAAC click reaction, metal-free click reaction, Diels–alder reaction, and Thiol-ene and thiol-yne reactions are the commonly employed click reactions in the fabrication of polymer nanocomposites [26]. Since the concept of this manuscript is mostly applications, the synthetic details are not included in the current paper. Additionally, different surface modification strategies have been developed, something which is also a very critical step in biosensing chemistry and design. Surface modification techniques significantly impact the nanocomposites’ structural and functional properties such as reactivity/chemical reactivity, biocompatibility/bioactivity, hydrophobicity, surface energy, dispersion/stability, and surface roughness. Surface modification can be achieved through different reactions with coupling agents and/or surface adsorption and polymeric molecules’ covalent or non-covalent bonding-based grafting. Along with such functionalization techniques, the reported polymer-nanocomposites-modified electrodes are very promising tools to enhance sensing capabilities in terms of high sensitivity and good selectivity for different types of targets such as drugs, heavy metals, pesticides, pathogens, etc. Polymer nanocomposites still hold a great strength in biosensor design since they provide variable morphologies and architectures on electrode surfaces such as films, vesicles, and dendritic structures [20]. While choosing the nanocomposites-based structural design, the main strategy is to add a nanofiller according to the need to give the final sensor system a targeted feature such as magnetism, fluorescence, electroconductivity, strength etc. The characteristics of a nanofiller considerably affects the properties of the polymer nanocomposites. The incorporation of a nano-scaled structure can add a new feature or improve an existing property. As demonstrated in Figure 1, in order to obtain a nanocomposite structure with magnetic properties, magnetic beads can be added to the composite structure, on the other hand quantum dots can be used as nano-modifier to prepare fluorescent polymeric nanocomposites. For the strength enhancement and hydrolytic stability, silica nanoparticles are a very appropriate choice and for their excellent mechanical stability, CNTs are very attractive nanofillers for the polymer nanocomposites. These unique properties, which are gained by adding nanofillers to polymer nanocomposites, significantly increase the analytical performance of the fabricated sensors. According to this approach, Table 1 represents the common nanofillers and their effect on polymer nanocomposite and advantages for the final properties of the fabricated biosensors. Here, the several forms of polymeric and biopolymeric nanocomposite films, the common nanofillers (Nanoclays, graphene, carbon nanoparticles, and quantum dots) and dendritic/vesicular polymeric nanocomposites from which they are made, and their use in biosensors and point-of-care systems are discussed in detail in light of recent advancements.

### 2.1. Nano-Clays

Nanoclay-incorporated polymers or copolymers have been a major focus of scientists working on biosensors. Nanoclays are well known and widely studied 2D nanomaterials in the surface modification of electrodes due to their mechanical and thermal stabilities, inert chemical structure, and unique and varying morphology [33,34]. According to the literature, the first combination of polymer and clay structures was reported by Blumstein in 1965 [35]. After demonstrating the increased thermal stability of polymers with this study, many nanocomposites have been produced to improve mechanical and structural properties. Owing to the remarkable advancements in material science, clay-reinforced polymer composites have been successfully adapted to analytical sciences. For instance, Emre et al. prepared polymethylmethacrylate (PMMA) layered silicate nanocomposites and evaluated their combinational use with conducting polymers with the name of poly(4-(2,3-dihydrothieno[3,4-b][1,4]dioxin-5-yl)-7-(2,3-dihydrothieno[3,4-b][1,4]dioxin-7-yl)-2-benzyl-1H-benzo[d]imidazole) (poly(BIPE)) as an immobilization platform for a glucose biosensor. This study demonstrated the prepared nanocomposite as a suitable matrix to protect enzyme molecules and provide proper surface chemistry for biomolecule attachment due to the aromatic groups of the conjugated polymer [36]. Another study regarding clay–polymer nanocomposites showed that a biodegradable polymer polyglycolide (PGA) and natural silicate montmorillonite composites could be applied as a coating material on electrode surfaces. Pyranose oxidase as a model enzyme was immobilized to the composite matrix, and the proposed biosensor was used for glucose detection in beverages without samples pretreatment [37]. Sarkal et al. developed a biopolymer-clay nanocomposite-based pesticide biosensor. The composite film comprising chitosan biopolymer and montmorillonite provided an eco-friendly immobilization matrix for acetylcholinesterase enzyme for organophosphorus pesticide detection with excellent sensing performance [38].

### 2.2. Graphene

Due to their excellent thermo-mechanical and electrical performance characteristics, graphene or graphene oxide is another widespread additive of polymer nanocomposites used to develop biosensors. Qiu et al. demonstrated the application of chitosan–ferrocene/graphene oxide nanocomposite film as an immobilization platform for a glucose oxidase enzyme. The developed sensor showed a fast response, high stability, good linearity, and sensitivity due to its multi-component structure and the redox mediator ferrocene group [39]. Graphene–polymer composite-based studies were used at the electrochemical sensor level and in the point-of-care test format. A label-free paper-based electrical biosensor chip developed from poly(styrene)-b-poly(acrylic acid) (PS67-b-PAA27) polymer and graphene nanoplatelet composite was recently presented. In this biosensor chip, an anti-cortisol antibody was immobilized over the electrode surface, and a layer-by-layer assembly process was applied for cortisol detection in saliva samples. The graphene nanoplatelet and amphiphilic di-block copolymer composite-based immunosensor exhibited outstanding analytical performance and had great potential for in vitro diagnostics [40].

### 2.3. Carbon Nanoparticles and Quantum Dots

Among the nanoparticles, multi-walled carbon nanotubes (MWCNT) and quantum dots (QD) have become prominent in biosensing applications. Another representative study for nanocomposite made from fullerene (C_60_), MWCNT, polyethyleneimine (PEI), and polymer QDs has been recently reported by Jamei and co-workers. They aimed to show the synergy of each nanocomposite component for the design of an aptasensor to analyze thrombin protein. While the examples above are enzyme- and antibody-based, this study shows that nanocomposites can also be used for aptamer immobilization. The C_60_/MWCNTs-PEI/PQdot/APT aptasensor exhibited excellent analytical performance in terms of sensitivity, selectivity, repeatability, and stability owing to the successful combination of various materials in the nanocomposite which offers high electrical conductivity, high surface-volume ratio, higher sites for the better attachment of aptamers, and high and stable mechanical and chemical structure [41].

### 2.4. Dendrimers

Polymer nanocomposites can form film-like surface coating layers as well as dendritic structures. For instance, dendrimers are valuable candidates for electrode covering in terms of their characteristic properties to maximize attachment points and flexible and well-oriented binding sites for biomolecules. When the multi-point binding ability of dendrimers is combined with the power of nanomaterials, dendrimeric nanocomposites have great potential in the fabrication of bioinspired devices and biosensors with high sensitivity and stability. In Luo’s work, the harmonious branched tree-like structure of Poly(amidoamine) (PAMAM)-Au nanocomposite was prepared as a matrix for horseradish peroxidase enzyme immobilization. The proposed mediator-free biosensor was fabricated on a MWCNT-modified glassy carbon electrode. Owing to the three-dimensional network of the hybrid surface material, unique bioelectrocatalytic capabilities were reported [42]. Another dendrimer nanocomposite-based biosensor was developed by Shukla et al. using zirconia-polypropylene imine dendrimer (ZrO_2_-PPI) nanocomposite to modify screen-printed carbon electrode surfaces by electro-co-deposition. The prepared nano-platform provided a suitable and biocompatible matrix for urease enzyme with its protected activity and high stability [43]. Recently, an interesting application of organic–inorganic composite nanomaterials for point-of-care diagnostics has been reported by Ruiz-Sanchez et al. Their novel approach was to obtain one-dimensional nanochains carrying the unique self-assembling properties of polyamidoamine dendrimers and AuNPs and to investigate their potential use as labels in lateral flow assays (LFA). Their findings confirmed that the prepared gold dendrimer nanocomposites increased the sensitivity by 4-folds compared to traditional AuNP (20 nm)-based sensors. This dendrimer nanocomposite-based approach promises to overcome sensitivity issues, which is the main drawback of LFAs [44].

### 2.5. Polymer Vesicles

Polymers can be self-assembled into several forms, such as micelles, vesicles, mono- or multi-layers, and nanosized particles. After discussing the micelles-shaped dendrimeric nanocomposite-based biosensor studies, the polymeric vesicles or spherical polymeric nanostructures are evaluated. Polymeric materials allow the obtention of biomimetic vesicles such as polymersomes derived from the self-assembly of various block polymers to produce a nan-sized composite. Because the polymersomes are flexible structures in terms of easy surface functionalization with biomolecules and encapsulation of multiple substances, they represent promising tools for drug delivery, bioimaging, diagnostics, and biosensor applications [45]. Polymersomes greatly impact optical biosensing systems because they allow the encapsulation of different colored dyes or fluorescence molecules and the attachment of antibodies or aptamers on their surface. Recent studies on point-of-care diagnosis, mainly due to the urgent needs associated with the COVID-19 pandemic, have focused on increasing selectivity and sensitivity. For this purpose, our group used dye-loaded polymersomes as labels in the design of paper-based rapid test kits as alternatives to AuNPs in traditional LFAs. Different test designs, including dot-blot assay [46] and lateral flow assay [47] were prepared, and their analytical performance parameters were investigated compared to RT-PCR methods. In the pre-clinical studies, a very high correlation was obtained between the proposed platform and the data from the RT-PCR results, even at low viral loads. This application has presented a novel and valuable scientific approach providing an urgent and cost-effective design strategy for pandemic sensors that can be applied to similar epidemics. Additionally, in the designed polymersome-based dot-blot assay, we reported a reference study that shows a comparison between AuNP-based and dye-loaded polymersome-based spot tests. The proposed diagnostic assay exhibited 10-times better sensitivity than AuNP [46].

As previously mentioned, nano-additives can incorporate into polymeric structures by creating polymer vesicles formed by closing the amphiphilic block copolymeric spherical lamellar structures in the appropriate solvent. For instance, in a similar strategy to develop alternative labels for an immunosensor system, Fe_3_O_4_ nanoparticles loaded poly(ethylene glycol)-poly(lactic acid) (PEG-PLA) polymeric vesicles were synthesized to fabricate a sandwich-type electrochemical immunosensor for the detection of prostate-specific antigen (PSA) as a model analyte. In the design strategy, while PEG-PLA polymeric vesicles carried secondary antibodies, the primary antibody was immobilized onto a graphene sheet surface. The prepared immunosensor based on nanoparticle-loaded polymer vesicles exhibited low LOD, high sensitivity, and stability [48].

The basis of such interest in polymer-based nanocomposites is the ability to design various morphologies and compositions with any desired decoration. In the fabrication of polymer and inorganic hybrid nano-objects, increasing the morphological diversity has been the driving force. Starting from this point of view, Fan et al. demonstrated a combined technique for quick preparation of polymer-gold nanocomposites with different morphologies, including sphere, worm, and vesicles. The facile technology they proposed combined the polymerization-induced self-assembly technique with “host-guest” chemistry. For the “host-guest” complexation, β-cyclodextrin (β-CD) and adamantane (Ada) was used. Cyclodextrins are composed of hydrophilic outer parts and a hydrophobic inner cavity, which can form non-covalent inclusion complexes with a guest molecule. This complexation strategy was adapted to achieve AuNP-decorated polymer nanocomposites; AuNP-Polymer sphere nano-flowers, AuNP–polymer sphere nano-patterns, AuNP-Polymer nano-worms, and vesicles. Briefly, β-CD functionalized block copolymer nano-objects were first prepared, and then the structures “host” and “guest” induced quick interactions between β-CD and Ada, which allowed the co-self-assembly of AuNP–polymer composites [49]. Our previous works have adapted the biomimetic property of β-CD units to electrochemical sensor platforms and LFA tests for cocaine detection. With these studies, a poly(p-phenylene) β-cyclodextrin poly(ethylene glycol) (PPP-CD-g-PEG) polymeric structure was specifically synthesized, and the β-CD cavity of the polymer was used as a biorecognition surface due to its ability to form CD–cocaine inclusion complexes [50,51]. It is conceivable that β-CD and inorganic materials (Au, Ag, Si, Fe_2_O_3_)-decorated polymer composites may be designed with various morphologies and compositions for biosensor applications in the near future.

In the light of the above reports, it is possible to conclude that coupling polymer nanocomposites with biosensing systems offer significant possibilities for improving sensor performances. Some recently published works on polymer and biopolymer nanocomposites-based biosensors are summarized in Table 2.

## 3. Conducting Polymer Nanocomposites

The four valance electrons of some polymers’ constructive carbon atoms are not fully used up in covalent bonds. These polymers are well known as conjugated polymers in which the electron delocalization provides high charge mobility along their carbon backbones. The conjugated polymers can possess semiconducting features or metallic properties depending on the number and kind of atoms within the repeated polymeric units. Conjugated polymers can also be transformed into conducting polymers by doping processes that change the number of π-electrons [74]. Heeger et al. received the Nobel Prize in Chemistry for the discovery of the first conducting polymer (polyacetylene). Conducting polymers (CPs) exhibit remarkable features such as high mechanical, electronic, optical, and environmental stability and low operating temperature and are lightweight, offer a simple synthesis, and economical behavior [75]. These outstanding features have led to the fabrication of optical wires, gadgets, and biosensor devices, such as sensor chips for diagnostic and environmental monitoring purposes [76,77].

With growing interest in this subject, many research reports have focused on advancing the properties of CPs and their synthesis approaches. The formation of composite and nanocomposite by the addition of fillers has been widely recommended to enhance the physical and chemical features of CPs. A composite typically consists of two or more constituents in which each component carries its features to the final structural material. Using nanomaterials of different types and shapes as a reinforcing phase in the CPs matrix phase creates a conducting polymer nanocomposite (CPNC). Several methods to produce CPNCs include electrochemical encapsulation, colloidal dispersions, in situ polymerization with nanoparticles, and coating of inorganic polymers [78]. The properties of CPNCs can be tuned by varying the matrix and filler, which results in millions of combinations usable in different applications. Alternative carbon nanomaterial (CNMs) fillers such as single-walled and multi-walled carbon nanotubes, fullerenes, carbon nanofibers, nanospheres, and graphene have been extensively used for CPNCs preparation [21]. The unique features of carbon nanomaterials, such as their environmental stability, surface area, and other properties (physical, chemical, thermal, and electrical), make them unique materials for the twenty-first century. Substantial efforts have been allocated to produce CPNCs with superior fundamental and technological assets through CNM and CP combination [79].

Other than CNMs, metal nanoparticles (silver, gold, platinum, etc.) and their oxide forms have been employed to create CPNCs with advanced features due to their different compositions and dimensions [80,81]. Various techniques such as electrochemical or chemical methods [82], sonochemical methods [83], sol-gel techniques [84], ultrasonic irradiation [85], and photochemical preparation [86] have been actively used to incorporate metals or metal oxide fillers into the preparation of conducting polymer nanocomposites. During preparation, nanocomposites show increased electrical breakdown strength, melting temperature, magnetization, charge capacity, and adopted behaviors such as electrical conductivity, corrosion resistance, dielectric, and semiconductivity. These advanced properties make CPNCs great candidates for developing electric-based biosensing platforms.

As an active biosensing platform, CPNCs such as nanocomposites of polypyrrole (PPy) and polyaniline (PANI) conducting polymers have shown high biocompatibility with cells and biological tissues. These features influenced researchers to employ CPNCs in tissue engineering, bio-electrodes, drug delivery, and biosensors to detect biological and synthetic moieties [87]. It has been demonstrated that the electrochemical performance of screen-printed electrodes-based biosensors is highly enhanced following the use of porous carbon, given the latter’s great conductivity and surface area. Moreover, using sulfur and nitrogen to dope carbon enhances its electrocatalytic properties. Taking this advantage, a nanocomposite formed by N and S-doped carbon and the polymer poly3-((2,20:50,2″-terthiophen)-30-yl)-5-aminobenzoic acid (pTTABA) was successfully used for neurotransmitters (NTs) detection. The proposed pTTABA-based amperometric biosensor exhibited a detection range of 0.5 µM to 4.0 mM with a limit of detection (LOD) reaching 112 nM for lactate detection [88]. A similar study proposed a combination of heteroatoms (N and S)-doped porous carbon and 2,2′:5′,5″-terthiophene-3′-*p*-benzoic acid (TBA) to produce an enhanced electrochemical microfluidic system for NTs determination in human plasma. This biosensor had an NTs detection range of 0.05–130 nM coupled with a highly sensitive LOD of 34–44 pM [89].

In addition to CNM nanocomposites, metal nanocomposites such as Pt and Au with PANI and PPy conducting polymers have been prepared and applied in biosensing applications. An antifouling electrochemical biosensing platform was designed based on embedded AuNPs into the conducting polymer poly(3,4-ethylene dioxythiophene) (PEDOT). The AuNPs functioned as signal enhancers, whereas the PEDOT acted as an antifouling agent over the sensor’s surface [90]. Another electrochemical biosensor comprising 3-mercaptopropionic acid (MPA) capped PtNP-PPy nanocomposite film was developed for C-reactive protein detection. The electrodeposition of Pt nanofibers creates numerous heterogeneous nucleating sites in the polymeric matrix resulting in highly controllable geometrical conformities. This nanocomposite allowed for enhanced orientation and easy access to the interaction between the analytes and biomolecules, leading to the detection of αCRP at a LOD = 4.54 ng/mL [91]. A study examining a PtNPs/PPy-based biosensor for sulfite detection in alcoholic beverages demonstrated satisfactory and fast analytical performances (LOD = 12.4 nM) obtained within 3–5 s [92].

Natural clay is an exciting material for sensor surface modification due to the outstanding features of the material, such as its porosity, ion exchange ability, high stability, and, most importantly, availability and low cost [91,92]. Zheng et al. developed a glucose biosensor using PANI-montmorillonite clay particles–PtNPs nanocomposite for glucose oxidase anchoring via electrodeposition. The biosensor was tested over human serum, providing a broad detection range (10 µM to 1.94 mM) [93]. Erkmen et al. also reported the preparation of a tyrosinase enzyme inhibition-based biosensor for the dual detection of catechol and azinphos-methyl. This platform consisted of a nanocomposite (poly (3,4 ethylene dioxythiophene) and iridium (IV) oxide) and tyrosinase crosslinked through glutaraldehyde. The biosensor could successfully detect catechol and azinphos-methyl samples with a LOD of 17 nM and 2.96 µM, respectively [94]. Several targets can be detected through nanocomposite conducting polymer-based biosensors, including hormones, enzymes, nucleotides, chemicals, organic compounds, microorganisms, neurotransmitters, vitamins, lipids, proteins, etc. Some examples of the pertinent works are summarized in Table 3.

## 4. Molecularly Imprinted Polymer Nanocomposites

Molecular imprinting technology is of great interest in biomimetic molecular recognition. Molecularly imprinted polymers (MIP), in which a target-specific cavity is created using a template, are considered an essential alternative to natural antibodies in bioanalytical devices. Due to their low cost, flexibility, outstanding chemical stability, and high recognition ability, MIPs have been used to fabricate biosensors in numerous studies. These artificial receptors suffer from some drawbacks, including long response time, heterogeneous structure of binding cavity, diffusion rate, etc. The main reason for these limitations is slow binding kinetics arising from the bulky forms of MIPs such as monoliths, thin films, microspheres, etc. The binding efficiency decreases as the recognition sites remain inside the MIP structure. Also, the low surface area of a general MIP results in a limited amount of recognition sites. Low affinity caused by these problems in biosensing systems has been the main focus for transforming the binding event into a successful signal [140]. Hybrid approaches to obtain MIP nanocomposites have great perspectives on enhancing biosensing performance by overcoming the limitations of traditional imprinted polymers. In the hybrid-material strategy, a variety of functional nanomaterials could be used. For their synthesis, the thickness of MIP films is controlled by an inorganic core. Some formation methodologies of such core–shell MIP nanocomposites are controlled through living radical polymerization (CRP, reversible/addition/fragmentation, chain transfer polymerization (RAFT), or atom transfer radical polymerization (ATRP) [141]. The application of molecular imprinting technology in nanocomposites combines the unique advantages of MIPs, such as affinity, physical and chemical stability, low cost, etc., with optical and/or electrical properties of nano-scaled materials. This synergy makes MIP nanocomposites very feasible tools for biosensor construction. Since the amount of high affinity imprinted sites alone is not enough for the sensitivity of a biosensor, the enlargement of surface area and manipulating interfacial properties by nanomaterials are important approaches in sensing strategies. Additionally, molecular geometry is a crucial factor to improve the binding ability of MIPs. Hence the sensitivity of a biosensor is also directly controlled by the imprinted polymer nanocomposites [142]. To increase detection selectivity and sensitivity, CNTs, AuNP, graphene, QDs and, SiO_2_, noble metal, Fe_3_O_4_ nanoparticles are the common modifiers of MIPs [143,144]. According to the target-oriented sensing strategy, the expected function can be achieved by choosing one of these nano-modifiers to develop different analytical methods, including optical, fluorescence, and electrochemical. For instance, among the nanomaterial-MIP hybrid materials, QDs are typical nanostructures used in fluorescence-based sensing applications due to their photostability and size-dependent fluorescence spectra. Although different nanofluorophores can be used in MIP fluorescence sensors, QDs have received greater attention because they offer narrower emission and broader absorption spectra. According to reports, there are several developed QD–MIP-based sensing methods [143,145]. In addition to fluorescence property, magnetic features can be added to MIPs for the construction of electrochemical biosensors. For example, Fe_3_O_4_ nanoparticles has been reported as an appropriate candidate by providing easy and quick fabrication technology to a biosensor system. Thanks to their excellent properties such as stability, catalytic activity, non-toxicity, and high surface area, Fe_3_O_4_ nanoparticles have been utilized to improve the sensitivity and selectivity of biosensors. Hence, Fe_3_O_4_ nanoparticles provide increased sensitivity while MIP has a unique cavity for the target molecule, and this magnetic MIP nanocomposite creates a precise sensor surface [146]. Another idea for MIP-hybrid-based fabrication strategies has been to utilize graphene for better electron transfer, higher mechanical strength, and increased specific surface area. This approach improves electrochemical assays’ performance due to the graphene nanohybrid-based electrochemical signal amplification [142]. Similarly, to add a particular function to a biosensing surface, SiO_2_ nanobeads can be used to form nanolayers for enhanced sensing ability [147]; TiO_2_ nanoparticles to provide high surface area, improved adsorption of the target, and quick electrochemical response [148]; silver nanoparticles (AgNPs) to incorporate optical properties [149]; and MWCNTs to accelerate the electron transfer [140] could be developed.

In a recently published work on a sarcosine sensor for prostate cancer diagnosis, the advantages of MIPs were combined with silica nanoparticles, making the resulting MIP nanocomposite more stable, biocompatible, and highly competitive permeable [150]. Similarly, a MWCNT/imprinted polymer nanocomposite-based potentiometric sensor was designed for lactic acid detection in dairy products. The purpose of the MIP decoration with a nano-object is the enhancement of electrical conductivity between the electrode and MIP surface and to improve the sensitivity with a wide calibration range [151]. In another work, a graphene oxide-based molecularly imprinted nanocomposite was reported for bisphenol A detection via electrochemical measurements. The work reported a combined approach for preparing a 3D network composed of graphene oxide, β-CD, and polyacrylate. The 3D network of these covalently linked components provided a molecular imprint of bisphenol A and presented a selective, rapid, and cost-effective method [152].

AuNPs are promising materials that permit excellent optical and electrical properties in diagnostic science and technology. These properties provide unique added characteristics in the production of MIP–AuNP nanocomposites. AuNPs have been combined with MIPs as nanocomposite films or colloidal particles. They have also been used to form multi-composite structures with other nanoparticles such as CNTi graphene, TiO_2_, etc. The design of these types of AuNP-based nanohybrid materials has been the main development topic for optical, gravimetric, and electrochemical biosensors. In a recent study, AuNP/MIP nanocomposite thin films were used over the electrode surface to fabricate a chemiresistive sensor to detect hexanal gas, a lung cancer biomarker in exhaled breath. This hybrid sensor layer allowed a successful detection window for hexanal gas with a good selectivity [153]. As previously mentioned, multifunctional hybrid nanocomposites can be designed to obtain multi-hybrid sensors based on AuNP/MIPs combined with CNT, graphene, or TiO_2_ nanoparticles to add synergic and beneficial effects of these nanoparticles to the designed sensors, including enhanced electron or charge transfer, increased electrocatalytic activity, improved sensor response and sensitivity, good mechanical robustness and chemical stability [154]. Lian et al. have reported a representative work for such an application introducing an imprinted sensor surface based on chitosan–platinum nanoparticles/graphene–AuNPs. With an advantageous combination of self-assembly and electropolymerization techniques, this approach was applied to obtain an erythromycin sensor with improved analytical capabilities [155]. Table 4 reports the MIP-based hybrid sensors decorated with different nanoparticles.

## 5. Hydrogel Nanocomposites

**Figure 2:**
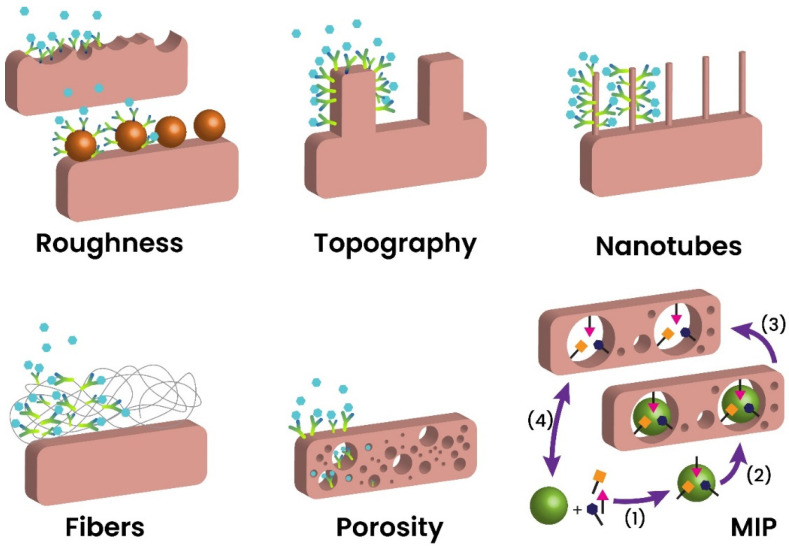
Typical 3D architectures used in biosensors, including roughness enhancement, topographical structures, fiber networks, etc. Molecularly imprinted polymers can be used in four steps: Assembly (1), deposition and polymerization (2), removal (3), and back to the initial step (4). Adapted with permission from Ref. [169]. ©2019, Elsevier B.V.

The growing trend of using biosensors for rapid diagnosis purposes has led to a greater focus on miniaturized biorecognition two-dimensional (2D) surfaces. However, these planner surfaces have exhibited limited analytical performances due to their narrow dynamic range, instability of the immobilized probes, longer response time, and low LODs [168]. Such constraints on the 2D structural biosensors eventually led to the design of three-dimensional (3D) biosensors. The newly designed biosensors successfully showed higher analytical performance, biocompatibility, enhanced selectivity, sensitivity, and flexibility for implantable devices (Figure 2).

The 3D polymeric networks in hydrogels can infuse a significant quantity of water and soluble molecules [170]. The weak mechanical strength of hydrogels is a considerable drawback limiting their performance where strength, elasticity, and endurance are highly demanded [171]. Other physicochemical criteria (diffusion, swelling, functional groups) should be closely considered when selecting materials for hydrogel manufacturing. Recent approaches in hydrogel optimization have led to the development of new hydrogel varieties such as nanocomposite hydrogels [172] and double network hydrogels [173]. The nanocomposite hydrogels containing various physically/chemically crosslinked nano-scaled structures among polymeric chains have shown novel properties and behaviors. Nanocomposite hydrogels can be created using different nanomaterials, including carbon-based nanomaterials, polymer NPs, inorganic/ceramic NPs, and metal NPs [174].

Creating nano/micro-sized matrices that benefit the unique properties of both hydrogels and nanomaterials is the main challenge in developing nanocomposite hydrogels. Typically, hydrogels’ flexible and 3D polymeric configuration can host different types of materials as a “guest” [175]. The gelation procedure of the final composite structure happens in any water-based or organic solution resulting in either hydrogel for aqueous media or organogel if made in organic media. Various natural polymers such as chitosan (CS) [176], cellulose [177], alginate [178], collagen [179], and lignin [180], as well as synthetic polymers, including poly(ethylene glycol) (PEG) [181], poly (N-isopropyl acrylamide) (PNIPAM) [182], poly(vinyl imidazole) [183], poly(vinyl alcohol) (PVA) [184], and poly(acrylic acid) (PAA) [185] show the ability to create hydrogels. The “host-guest” interaction between hydrogels and nanomaterials is formed through covalent and non-covalent bonding, such as hydrogen bonding, van der Walls forces, and electrostatic interactions [186]. The application of functionalized nanomaterials can significantly enhance the features of the final nanocomposite hydrogel, namely the mechanical properties and bioactivity [187]. On the contrary, by taking advantage of their chemical structures and forming π-π stacking interactions, non-functionalized nanomaterials (graphene and carbon materials) can also enhance some aspects of hydrogels compared with pure ones [175].

Other than organic-based nanomaterials, inorganic nanomaterials (nanoclays, ceramics, bioactive glass, metallic NPs) are actively utilized to produce nanocomposite hydrogels [174]. The implication of metal NPs and their oxide forms in the hydrogel structure has brought up attractive attributes such as magnetism, electrical and thermal conductivity, and antimicrobial activities. This makes these nanocomposite hydrogels great alternatives as sensors and conductive scaffolds in addition to other applications such as drug delivery [174].

Biosensors containing nanocomposite hydrogels as 3D material supports exhibit distinguishable performance and minimized platform cost. The unique structure of these hybrid hydrogels preserves the biological activity of the probe molecules by reducing steric hindrance and improving probe orientation and stability for enhanced analyte capturing. Introducing conductive materials into hydrogel matrices is a well-known approach for sensor applications due to their functionalities. Amongst conductive materials, graphene and carbon nanomaterials [188,189], nanocrystals [190,191,192], and conducting polymers [186,192,193] are mainly applied as conductive additives to hydrogels. The addition of conducting polymers (PPy and PANI) to the hydrogel matrices usually occurs along with electrochemical polymerization procedures. On the contrary, carbon nanotubes and graphene are generally incorporated into the matrices via various mixing methods.

Depending on the nature of the added conductive components, the charge transfer can be accelerated, and the signal made stronger [190,191,192]. For instance, Wang et al. developed a carbon–PPy hydrogel nanocomposite-based biosensor for acetaminophen detection. The addition of the porous carbon to the matrix enhanced the analytical performance of the sensor in acetaminophen determination at nanomolar levels (LOD = 1.2 nM) [193]. Xu et al. reported the development of a PANI conductive polymer-hydrogel 3D material for xanthine detection. The purine base was detected by measuring the produced hydrogen peroxide [194]. Zhao et al. also reported the development of an animal skin-inspired conductive hydrogel-based biosensor containing polydopamine-AgNPs, PANI, and polyvinyl alcohol for skin sensing and wound-dressing for diabetic patients [195]. Table 2 shows the components of biosensors containing conducting polymers, carbon, and graphene nanocomposite hydrogels. The sensing mechanism of these biosensors strongly depends on the electrochemical charge transfer as the added nanocomposite is beneficial for charge transport.

**Figure 3:**
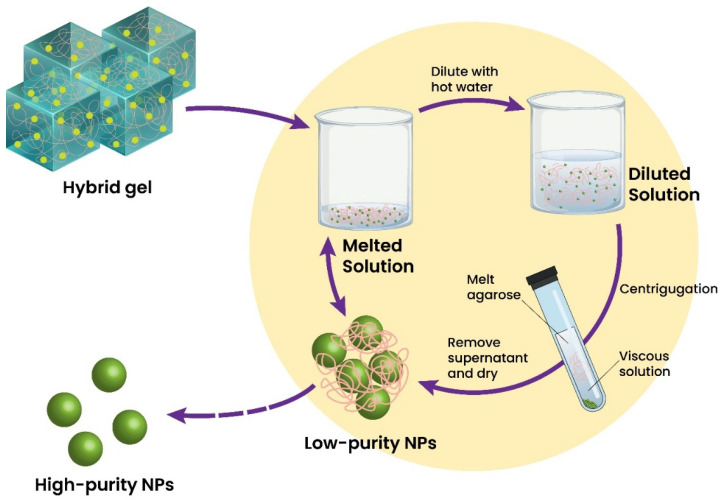
TiO_2_/agarose hybrid gel and the recycling and recovery of TiO_2_ nanoparticles through heating and separation techniques. Adapted with permission from Ref. [201]. ©2017, Royal Society of Chemistry.

A limited number of inorganic nano-scaled materials can be used directly in sensing platform formation. Inorganic components have specific characteristics, making them very competitive to reach a defined purpose and function. Therefore, these materials are generally used as primary or secondary components. On the contrary, inorganic nanomaterials can be widely utilized as additives to enhance the analytical performance of sensors. Forming homogeneous dispersions of inorganic materials is hard to achieve, whereas using a strong mixing procedure could denaturalize the hydrogel structure. If inorganic additives are effectively dispersed into hydrogels, the obtained sensor can exhibit steady signal transduction and stable performances. Among the wide range of inorganic materials used to improve performance, nano-scaled silica [196], titanium oxide [197], quantum dots [198], and organosilicates [199] are preferred for the functionalization of hydrogels. For example, Huang et al. reported using a magnetic Fe_3_O_4_ nanoparticles-embedded hydrogel to form a fiber-optic glucose biosensor. The proposed biosensor demonstrated temperature-adjusted glucose-sensing within 50 to 700 mg/dL and an LOD = 8.3 mg/dL [196]. Cui et al. attempted the creation of a TiO_2_-chitosan and Au nanorods–SiO_2_ NPs nanocomposite hydrogel embedding acetylcholinesterase for organophosphate pesticides sensing. This biosensor showed a linear range of 18 Nm–13.6 μM and LODs of 5.3 nM and 1.3 nM for dichlorvos (DDVP) and fenthion, respectively [200]. Titanium dioxide nanoparticles (TiO_2_) can be used actively as an additive in hydrogel matrices without losing their photocatalytic activity [201]. TiO_2_ nanocomposite hydrogel can be recycled through heating and separation methods allowing the hybrid gel to be simply remodeled into new hydrogel forms of different shapes and sizes (Figure 3).

Other inorganic nanomaterials such as QDs [202,203,204], noble metal [198,205], and magnetic nanoparticles [206] took part in many research areas. Cadmium Selenide QDs nanocrystals were combined with PEG-based hydrogels and successfully applied for phenol detection [202]. Enzyme encapsulated cadmium telluride QD-based hydrogels with a biocatalysis unit and a fluorescence signaling unit was utilized as a multifunctional material to develop optical biosensors [203]. Magnetic nanoparticles in sensors and biosensors are in high demand due to the limited accessible number of candidate materials. Jia et al. created an aldehyde biosensor based on a responsive photonic hydrogel formed through self-assembled carbon-Fe_3_O_4_ NPs and in situ photopolymerized polyacrylamide hydrogels [206]. Table 5 summarizes the notable features of the inorganic nanocomposite hydrogel-based biosensors introduced in this section. Despite organic nanocomposites (polymeric and carbon-based nanocomposites), the mechanism of electrochemical sensing varies depending on the type of inorganic materials. Still, photochemical identification is retained for the hydrogels containing inorganic additives.

## 6. Conclusions and Future Perspectives

Biosensors have become a standard analytical tool in various fields, especially healthcare and biomedicine. Despite their several advantages, many challenges such as dynamic range, stability, chemical reactions, etc., obstruct their applications. Consequently, great efforts are focused on developing novel materials and structures to enhance analytical features to reach commercialization.

Polymers and nano-scaled materials have many advantages that can answer and overcome many of the issues faced before. Their combination for the creation of nanocomposite materials provides an exciting opportunity to complement both materials and bypass the disadvantages seen for each material alone.

One of the most critical requirements in polymer nanocomposites synthesis is the creation and then dispersion of the homogenous matrix, which plays an essential key role in the composite’s physical and chemical features. Unfortunately, most applied processes are not feasible economically or cannot deal with poor interfacial adhesion and agglomeration of nanoparticles. Some approaches, such as layer-by-layer assembly and electrospinning, could be applied to create perfectly homogenous polymeric matrices but are not suitable for scaling up or commercialization. The melting process is economically achievable among all the alternative methods, but the final polymeric matrices mostly show poor and non-homogeneous dispersion. Reinforcing the melting process with high shear mixing methods like twin-screw extruding might guarantee adequate dispersion.

Protection and proper orientation of the functional groups in nanocomposites is another challenge that can be overcome by 3D or 4D printing to further enhance the final product’s mechanical properties. It is also important to solve the altered rheological properties resulting from the polymer chain flexibility restriction after nanocomposite integration in the polymer matrices. Hence, selecting nanocomposites with suitable sizes and shapes may provide decent interactions between the polymer and nanomaterials.

Modifying the polymers by changing their morphology, optimum aspect ratio, surface roughness, or introducing functional groups can prevent the possible structural defects related to the synthesis process conditions (temperature, pressure, density, and speed rate). For example, under high temperatures, the chance of having a structure with fewer hollows and porosities is higher. However, these conditions can impose a high cost and waste of materials that must be considered during the design and manufacturing phase. Consequently, developing cost-effective synthesis approaches with commercialization prospects will be interesting.

We have discussed polymer and nano-scaled nanocomposite materials to develop sensing platforms throughout the current review paper. The diversity of these materials show important advances in improving various sensing features such as sensitivity, selectivity, LOD, storage, etc. We hope that these advances will significantly bridge the gap between R&D-based experiments and approved clinical and commercial applications.

## Figures and Tables

**Table 1:** The effect of several nanofillers on the improved properties of polymeric nanocomposites and their sensors.

Nanofiller	Polymeric Composite	Effect for the Fabricated Composites	Advantages of the Sensors	Ref.
Nanoclay	OMMT/PLA	Improved thermal and mechanical property	Improved surface morphology and surface reflectance, modified optical properties	[27]
Graphene	GC-COOH	Electroactivity	High electroactivity and easy assembly, high sensitivity,	[28]
CNT	Chitosan modified by ferrocene and CNT	Increased surface area and decreased effective distance between mediator molecules	Increased recorded analytical signal, and measurement sensitivity	[29]
PAMAM dendrimer	PAMAM-PPy	Functionality and increased quantity and homogenous distribution of attached biomolecules	Efficient electron transfer, reversible redox system, and simple reaction procedure	[30]
Oleic acid-modified MNPs	Magnetic cyclodextrin vesicles	Magnetic property	Higher sensitivity	[31]
Nano rod and Quantum Dot	TiO_2_ Nanorod/TiO_2_ Quantum Dot/Polydopamine	Strong light absorption and excellent photocatalytic activity	Stronger photoelectric response under visible light	[32]

PLA: Polylactide, OMMT: Organically modified montmorillonite, GC-COOH: Carboxylated chitosan-functionalized nitrogen-containing graphene, CNT: Carbon nanotube, PAMAM-PY: poly(amidoamine) dendrimers and polypyrrole film, MNPs: Magnetic nanoparticles.

**Table 2:** Recently developed biosensors based on polymer–biopolymer nanocomposites.

Polymer/ Biopolymer	Nanocomposites	Target Analyte	Limit of Detection	Sensor Type	Ref.
Amphiphilic polymer	Polymeric-coated Fe-doped ceria/gold	2,4-Dinitrophenol	0.45 μg/mL	Optical biosensor	[52]
Chitosan	MT/Chitosan	Organo-phosphorus pesticide	0.448 µg/L	Electrochemical	[38]
	GO/Chitosan	Amine vapors	MA: 2.2 ppm DMA: 2.6 ppm TMA: 1.3 ppm	QCM	[53]
	AuNP/graphene/ chitosan	H_2_O_2_	1.6 μM	Electrochemical	[54]
	GNRs/chitosan	Sarcosine	0.001 μM		[55]
	CuS/NSC	Glucose	2.72 μM		[56]
	3D-NPZnO/Chitosan		0.2 mM		[57]
	AuNP-Chitosan-IL	Malathion	0.68 nM		[58]
CMC	rGO-CMC	NO and H_2_O_2_	0.37 μM and 0.08 μM		[59]
G4-PAMAM	AuNP/G4-PAMAM dendrimer	Insulin	0.5 pM	Surface plasmon resonance	[60]
	MWCNTs/G4-PAMAM dendrimer	Cellular prion proteins	0.5 pM	Electrochemical	[61]
PABS	SWCNT/PABS	Hg(II)	0.06 μM		[1]
PAMAM	GO/PAMAM dendrimer	CCRF-CEM cell	10 cells/mL		[62]
	PtNP/PAMAM dendrimer	H_2_O_2_	141 μM	Electrochemiluminescence	[63]
PAN	MT/PAN	Glucose	2.4 mM	Electrochemical	[64]
	AgNO_3_/PAN nanofiber	Triglyceride	10.6 mg/dL		[65]
PEI	Au@Ti_3_C_2_@PEI-Ru(dcbpy)_3_^2+^	SARS-CoV-2 RdRp Gene	0.21 fM	Electrochemiluminescence	[66]
PGMA and PEI	Ag-PEI-cPGMA	*E. coli*	-	Raman scattering	[67]
poly(N-methylaniline)	poly(N-methylaniline)-Ce_2_(WO_4_)_3_@CNT	Cd^2+^	0.11 nM	Electrochemical	[68]
Polyrhodanine	Graphene oxide/Fe_3_O_4_/ polyrhodanine	Doxorubicin	0.008 μM		[69]
PPI	AuNP/PPI dendrimer	ssDNA	0.05 nM		[70]
PS and PANI	Au/PS/PANI	Glucose	12 μM		[71]
PS-b-P4VP	AuNP/PS-b-P4VP	Human IgG	1.1 nM	Surface plasmon resonance	[72]
PVP/PVA/PAM	Ti_3_C_2_ MXene/PVP/ PVA/PAM	Dopamine	0.2/0.3 /0.1 × 10^−4^ mol/L	Electrochemical	[73]

3D-NPZnO: 3D-nanoporous Zinc oxide, AuNP: Gold nanoparticle, CCRF-CEM cell: Human acute lymphoblastic leukemia, Ce_2_(WO_4_)_3_: Cerium tungstate, CMC: carboxymethyl cellulose, CuS/NSC: N and S co-doped chitosan polymer, DMA: Dimethylamine, G4-PAMAM: Fourth-generation polyamidoamine dendrimer, GNRs: Graphene nanoribbons, GO: Graphene oxide, IgG: Human gamma globulin, IL: Ionic liquids, MA: Methylamine, MT: Montmorillonite, MWCNT: Multi-walled carbon nanotubes, PABS: Poly (m-amino benzene sulfonic acid), PAN: Polyacrylonitrile, PANI: Polyaniline, PEI: Polyethyleneimine, PGMA: poly(glycidyl methacrylate), PPI: Poly(propyleneimine), PS: Polystyrene, PS-bP4VP: poly(styrene-b-4-vinylpyridine), QCM: Quartz crystal microbalance, rGA: Reduced graphene oxide, SWCNT: Single-walled carbon nanotube, TMA: Trimethylamine.

**Table 3:** Various nanoparticles/conducting polymer-based nanocomposites and their characterization techniques, target analyte, and detection limit.

Conducting Polymer	Nanocomposites	Target Analyte	Limit of Detection	Sensor Type	Ref.
APS	FcA/APS	H_2_O_2_	2.07 µM	Electrochemical, Fluorescence	[95]
P3ABA	Pt@rGO/P3ABA	Glucose	44.3 µM	Electrochemical	[96]
		Cholesterol	40.5 µM		
PABA	G/PABA	Acetylcholine	2.3 µM	Electrochemical	[97]
PANI	Au NPs/PANI	Prostate-specific antigen	0.085 pg/mL		[98]
		Dopamine	0.1 µM		[99]
		Melamine	1.39 pM		[100]
	Cu-BTC/PANI	*E. coli*	2 cfu/mL		[101]
	G/PANI	Dopamine	1.98 pM		[102]
		Anthracene	4.4 nM		[103]
	GO/PANI	DNA	20.8 fM		[104]
	NiCo_2_O_4_/PANI	Glucose	0.38 µM		[105]
	MWCNTs/PANI	Cholesterol	0.01 mM		[106]
		Cardiac troponin T	0.04 pg/mL		[107]
	NiO/CuO/PANI	Glucose	2 µM		[108]
	NiO/PANI		0.06 µM		[109]
	Pt NPs/PANI	Uric acid	0.001 mM		[110]
		Cholesterol	0.3 mM		[110]
		Triglyceride	0.2 mM		[110]
	ZnO/MWCNTs/PANI	Glucose	0.1 mM		[111]
	G/PANI	Estradiol	0.02 ng/mL	Immunosensor	[112]
PANI@PPY	MWCNTs/PANI@PPY	H_2_O_2_	0.1 µM	Electrochemical	[113]
PDA	MWCNTs/PDA	Cholesterol (Ch) (Ch oxidase/SPE)	1.5 µM	Electrochemical	[114]
PEDOT	Au NPs/PEDOT	Triglyceride	89 µM		[115]
		CA15-3	35.64 mU/mL		[90]
	CNTs/PEDOT	Dopamine	20 nM		[104]
	AuNPs-MWCNT/PEDOT	Catechol	0.11 µM		[116]
		Laccase	12.26 µM		
	CNTs/PEDOT	*Mycobacterium tuberculosis*	0.5 fg/mL		[117]
	Fe_2_O_3_/PEDOT	Carcinoembryonic antigen	-	Electrochemical paper-based	[78]
	GO/PEDOT	Dopamine	90 nM	Electrochemical	[118]
	MWCNTs/PEDOT	Magnolol	3 nM		[119]
	PtNPs-PEDOT	Glucose	1.55 µM		[120]
	RGO/PEDOT	Dopamine	78 fM		[121]
			39 nM		[122]
	ZrO_2_/PEDOT	Vitamin B2	0.012 µM		[123]
		Vitamin B6	0.2 µM		
		Vitamin C	0.45 µM		
PIn-5-COOH	MWCNTs/PIn-5-COOH	α-fetoprotein	0.33 pg/mL	Immunosensor	[124]
POT	Au NPs/POT	Glucose	0.2 mM	Electrochemical	[125]
PP3C	GO/PP3C	Glucose	0.05 mM	Electrochemical	[126]
PPy	Ag@ZnO/PPy	Xanthine (X)	0.07 µM		[127]
	Au NPs/PPy	Carcinoembryonic antigen	1.6 × 10^−7^ ng/ml	Immunosensor	[128]
		Dopamine	1.5 × 10^−8^ M	Electrochemical	[129]
		Serotonin	1.0 × 10^−9^ M		
		DNA	8.4 × 10^−12^ M		[130]
	CeO_2_-NRs/Ppy	DNA from *Salmonella*	0.29 vM		[131]
	Ferrocene/PPY	*M. tuberculosis*	0.36 aM		[132]
	G/PPy	Adenine	0.02 µM		[133]
		Guanine	0.01 µM		
	MWCNTs/PPy	6-mercaptopurine	0.08 µM		[134]
		Glucose	0.43 µM		[135]
	NiO/PPy	Glucose	0.33 µM		[136]
	NiCo_2_O_4_/PPy		0.22 µM,		[137]
	ZnFe_2_O_4_/PPy		0.1 mM		[138]
PTBA	S, N-doped carbon/PTBA	Neurotransmitters	0.034 nM	Electrochemical	[89]
PVDF	Carbon black/PVDF	IL-8 biomarker	3.3 fg/mL	Immunosensor	[139]

AgNPs: Silver nanoparticles, APS: Amino-polyethersulfone, Au NPs: Gold nanoparticles, BTC: 1,3,5-benzene tricarboxylic acid, CeO_2_: Cerium oxide, CNTs: Carbon nanotubes, Cu: copper, FcA: ferrocene carboxylic acid, Fe_2_O_3_: Iron (II, III) oxide, G: Graphene, GO: Graphene oxide, MWCNT: Multi-walled carbon nanotubes, NiCo_2_O_4_: Nickel cobaltite, NiO: Nickel oxide, P3ABA: Poly(3-aminobenzoic acid), PABA: Poly(3-amino-benzylamine-*co*-aniline), PANI: Polyaniline, PDA: Polydopamine, PEDOT: Poly(3,4-ethylene dioxythiophene), PIn-5-COOH: Poly(indole-5-carboxylic acid), PP3C: Poly(pyrrole-3-carboxylic acid), PPY: Polypyrrole, Pt NPs: Platinum nanoparticles, PTBA: Poly 2, 2′:5′, 5″-terthiophene-3′-*p*-benzoic acid, PVDF: Polyvinylidene fluoride, RGO: Reduced graphene oxide, TiO_2_: Titanium dioxide, ZnO: Zinc oxide, ZrO_2_: Zirconium dioxide.

**Table 4:** Some nanomaterials combining MIP architectures for biosensor applications.

MIP Nanocomposite	Target Analyte	Limit of Detection	Sensor Type	Ref.
AgNWs-MIPs	Lactate	0.22 μM	Electrochemical	[156]
AuNPs-GO-MIP	BRCA1 gene	2.53 fM		[157]
bAu@mSiO_2_@MIP	Enrofloxacin	1.5 nM	Optical biosensor	[141]
CdS/CdTe QDs/MIP	BSA	0.5 μM		[158]
Fe_3_O_4_-MIP	Tributyltin	5.37 pM	Electrochemical	[159]
fMWCNTs-MIP	Norfloxacin	1.58 nM		[160]
GO-MIP	Cholesterol	0.1 nM		[161]
MIP@CdTe QDs	Lysozyme	3.2 μg/mL	Optical biosensor	[162]
MWCNT-MIP	Chlorpromazine	0.29 nM	Electrochemical	[163]
MWCNTs-Chit-MIP	HCV antigen	1.67 fg/mL		[164]
PGr/CdTe QDs/Fe_3_O_4_@SiO_2_/MIP	Cefoperazone	0.09 μg/L	Optical biosensor	[165]
SiC-MIP	Loratadine	0.15 μM	Electrochemical	[166]
SMoSe_2_/NSG/Au/MIPs	Dopamin	0.02 μM		[167]

bAu@mSiO_2_@MIP: Multibranched gold−silica−molecularly imprinted polymer, BRCA1: Breast cancer susceptibility gene, BSA: Bovine serum albumin, GO: Graphene oxide, HCV: Hepatitis C virus, MWCNT: Multi-walled carbon nanotube, SiC: Silicon carbide nanoparticles.

**Table 5:** Collection of some of the pertinent nanocomposite hydrogel applications for biosensing.

Additives	Analyte	Sensing Method	LOD	Ref.
Ag NPs@PEG	Fe^3+^	Fluorescence	45 µM	[198]
	Thiosulfate		60 µM	
ALP	Vanadium	Electrochemical	230 nM	[207]
Aptamer@carboxylated PPy nanotubes	Dopamine	Electrochemical	1.0 nM	[208]
Au nanorod@SiNP-doped TiO_2_-chitosan	Dichlovos	Electrochemical	5.3 nM	[200]
	Fenthion		1.3 nM	
Au NPs	Glucose	Electrochemical	370 nM	[209]
AuNCs modified DNA-aptamer	Progesterone (P4)	Electrochemical	1.0 ng/mL	[210]
Carbon dots	microRNA-21 in breast cancer cells	Fluorescence	0.03 fM	[211]
Carbon-encapsulated Fe_3_O_4_ NPs@PAAm	Formaldehyde	Colorimetric	-	[206]
CNCs	Strain sensor	Electrochemical	-	[212]
Co_3_O_4_@GO	Glucose	Electrochemical (Voltammeter)	250 µM	[190]
Fe_3_O_4_@SiO_2_(F)@meso-SiO_2_ nanoparticles	glucose	Fluorescence quenching	0.46 mM	[196]
GO	Glucose	Optical, Electrochemical	25 µM	[213]
	Biochemical oxygen demand	Fluorescent	0.4 mg	[214]
	Antibiotic		25 mg/L	[189]
	Strain sensor	Electrochemical	-	[215]
GO/PANI	BSA	Near-infrared light-responsive electrochemical	15 nM	[216]
Ionic liquid hydrogel-Au nanoballs-MoSe_2_	Carcinoembryonic antigen (CEA)	Photo-electrochemical (Photocurrent)	11.2 nM	[121]
Ionic liquid-AuNP and ZnCdHgSe QDs	Human epididymis protein 4 (HE4)		15.4 nM	[217]
Laponite@VBA	Glucose	Electrochemical (Current)	200 mM	[199]
Lignocellulose nanofibers/LC	Strain and pressure sensor	Electrochemical	-	[218]
MSA-capped CdTe QDs	Dopamine	Fluorescent	50 nM	[203]
N-doped porous carbons	Acetaminophen	Electrochemical	1.2 nM	[193]
NiCo_2_O_4_ nanoflowers@3D nitrogen-doped graphene	Glucose	Optical, Electrochemical	390 µM	[192]
	Hydroperoxide		136 µM	
PANI	Ascorbic acid	Spectrometric (Infrared)	1.28 mM	[219]
	Dopamine		44 µM	
	Uric acid	Electrochemical	46 µM	
	Xanthine	Optical	9.6 nM	[194]
Pd@Au NPs	microRNA let-7a (miRNA let-7a)	Electrochemiluminescence	1.49 fM	[220]
PDA@Ag NPs	Epidermal	Electrical	-	[195]
PEDOT/PSS	Strain sensor	Electrochemical	-	[221]
PEG@ CdSe/ZnS QDs	Phenol	Fluorescence quenching	1.0 mM	[202]
PEG@Ag NW	neuronal stem cells (NSC)-derived neural differentiation	Fluorescent	Neurite length (30–140 mm)	[205]
Plasmonic silver nanocubes	Glucose	Optical	2.29 mM	[222]
PPy		Electrochemical (Amperometry)	4.0 µM	[223]
Pt NPs@3D graphene		Electrochemical (Voltammeter)	5.0 mM	[191]
Pt NPs@PANI	Triglycerides	Electrochemical (Amperometry)	70 µM	[224]
	Lactate		60 µM	
	Glucose		200 µM	
	Uric acid		70 µM	[110]
	Cholesterol		300 µM	
	Triglycerides		200 µM	
SA-B-DAPPy	Strain sensor	Electrochemical	-	[225]
SiO_2_	Strain and pressure sensor		-	[226]
TEGO	Human-body motion and glucose	Electrochemical and mechanical	200 nM	[227]
Thioglycolic acid-QDs and N-Acetyl-l-cysteine-QDs	Fe^3+^ ion	Optical, Fluorescent	14 nM	[228]

Ag NPs: Silver nanoparticles, Ag NW: silver nanowire, ALP: Alkaline phosphatase, Au NPs: Gold nanoparticles, BSA: Bovine serum albumin, CdSe: Cadmium selenide, CdTe: Cadmium telluride, CNCs: Cellulose nanocrystals, Co_3_O_4_: Cobaltosic oxide, Fe_3_O_4_: Iron(II, III) oxide, GO: Graphene oxide, LC: Lignin-based carbon, MoSe_2_: Molybdenum diselenide, MSA: Mercaptosuccinic acid, NiCo_2_O_4_: Nickel cobaltite, PAAm: poly(acrylamide), PANI: Poly(aniline), PDA: Polydopamine, PEDOT/PSS: Poly(3,4-ethlenedioxythiophene)/poly(styrenesulfonate), PEG: Poly(ethylene glycol), PPy: Poly(pyrrole), Pt NPs: Platinum nanoparticles, PVA: polyvinyl alcohol, QDs: Quantum dots, SA-B-DAPPy: Sodium alginate (SA) and dopamine functionalized polypyrrole, Si NPs: Silicon nanoparticles, SiO_2_: Silicon dioxide, TEGO: thermally exfoliated graphene oxide, TiO_2_: Titanium dioxide, VBA: vinylbenzyl triethylammonium chloride, ZnCdHgSe: Zinc cadmium mercury and selenium, ZnS: Zinc sulfide.

## Data Availability

Not applicable.
